# Association of type 2 diabetes with osteoporosis and fracture risk: A systematic review and meta-analysis

**DOI:** 10.1097/MD.0000000000041444

**Published:** 2025-02-07

**Authors:** Yang Cao, Bo Dong, Yue Li, Ying Liu, Li Shen

**Affiliations:** a The First School of Clinical Medicine, Shaanxi University of Chinese Medicine, Xianyang, China; b Department of Orthopedics, Xi’an Honghui Hospital, Xi’an Jiaotong University, Xi’an, Shaanxi, China; c Institute of Chinese Materia Medica, China Academy of Chinese Medical Sciences, Beijing, China; d Institute of Basic Theory for Chinese Medicine, China Academy of Chinese Medical Sciences, Beijing, China.

**Keywords:** bone, diabetes mellitus, fractures, osteoporosis, systematic review and meta-analysis, type 2

## Abstract

**Background:**

Osteoporosis, a systemic skeletal disease characterized by low bone mass and increased fracture risk, poses significant social and economic challenges globally, while type 2 diabetes mellitus (T2DM), a prevalent metabolic disorder, has been linked to complex effects on bone health, including contradictory findings on its relationship with osteoporosis and fracture risk.

**Methods:**

We searched PubMed, Embase, Cochrane, and Web of Science Library to identify observational studies investigating whether people with T2DM have a higher risk of osteoporosis or fracture than people without diabetes. The time limit for literature retrieval was from the establishment of the database until March 2023. The quality of the studies was assessed using the Newcastle-Ottawa Scale and Agency for Healthcare Research and Quality checklist. The meta-analysis was conducted using Stata 15, and a random-effects model was used if I^2^ was > 50%. The Egger test was used to assess publication bias.

**Results:**

The results demonstrated that people with T2DM have a higher risk of osteoporosis. (relative risk, 1.841; 95% confidence interval, 1.219–2.780; *P* = .004). Similar results were demonstrated for fractures (relative risk, 1.21; 95% confidence interval, 1.09–1.31; *P* < .001). However, the results of the subgroup analysis showed no significant correlation between T2DM and fractures in univariate analysis, cross-sectional studies, Asia, Europe, Oceania, and vertebral fractures. However, a significant correlation was found in other subgroup analyses.

**Conclusions:**

Osteoporosis and fractures are significantly associated with T2DM.

## 1. Introduction

Osteoporosis is a systemic skeletal disease characterized by low bone mass and microarchitectural deterioration of the bone tissue, leading to enhanced bone fragility and increased fracture risk.^[1]^ As the world’s population ages, the social and economic burdens of osteoporosis are steadily increasing.^[2]^

Diabetes mellitus type 2 (T2DM) is a major health burden worldwide owing to its high prevalence, morbidity, and mortality. The global diabetes prevalence in 20 to 79 year olds in 2021 is estimated to be 10.5% (536.6 million people), rising to 12.2% (783.2 million) in 2045. Diabetes prevalence was highest in those aged 75 to 79 years.^[3]^

The relationship between T2DM, osteoporosis, and fractures is disputed. Nevertheless, it has been demonstrated that patients with T2DM have an average or higher bone mineral density (BMD) than age-matched controls.^[4]^ Inconsistent findings have been reported regarding sex differences in the association of diabetes mellitus with the excess risk of osteoporosis and fracture. A previous meta-analysis^[5]^ indicated that the female sex (odds ratio (OR) = 2.973, 95% confidence interval (CI): 1.678–5.268) was a risk factor for T2DM combined with osteoporosis. In some studies, diabetes was significantly associated with an increased risk of fractures,^[6]^ hip fracture,^[7,8]^ and vertebral fracture.^[7]^ In contrast, other studies have found no association between diabetes and fractures,^[9]^ hip fracture,^[10]^ and vertebral fractures. After developing T2DM, BMD has been found to increase in some studies.^[4]^ Paradoxically, despite elevations in BMD, which should portend increased skeletal strength, men and women with T2DM are at an increased risk of fracture.^[11,12]^ However, research on the risk of fracture and osteoporosis in T2DM has shown an opposite.^[5–10]^ To provide a more comprehensive analysis, update the research content, and improve the evidence, the present study conducted a meta-analysis and systematic review of the relationship between T2DM and the risk of osteoporosis and fractures.

## 2. Methods

In alignment with the Preferred Reporting Items for Systematic Reviews and Meta-Analysis guidelines,^[13]^ we conducted this study. Our research protocol was registered in PROSPERO (CRD42023462942).

### 2.1. Search strategy

Publications indexed in Embase, PubMed, Web of Science, and Cochrane Library from inception to March 2023 were independently searched by 2 investigators (YC and YL). Search terms were derived from terms related to “Diabetes Mellitus, Type 2,” “Osteoporosis” and “Fractures, Bone” without any restrictions on the language and time of publication. Detailed full-search strategies are provided in Table S1, Supplemental Digital Content, http://links.lww.com/MD/O345. To avoid omission of relevant literature, the reviewers conducted multiple reviews of all relevant citations. Moreover, manual searches of the reference lists of relevant studies were performed to identify additional eligible studies.

### 2.2. Inclusion criteria and exclusion criteria

According to the 2006 World Health Organization guidelines, T2DM is diagnosed as fasting blood glucose ≥ 7.0 mmol/L or 2 hour plasma glucose ≥ 11.1 mmol/L.^[14]^ Individuals without type 1 diabetes on diabetes medication were classified as having T2DM.^[15]^ Patients with T2DM were included in the study regardless of treatment, blood glucose status, or intervention. The control group comprised individuals without T2DM. The outcome was the presence of a fracture or osteoporosis. The types of studies were not limited, and cohort, case–control, or cross-sectional studies were acceptable. The exclusion criteria comprised articles with unclear data or obvious inconsistencies and patients with other types of diseases not as a single outcome.

### 2.3. Data extraction

Data extraction was carried out independently by 2 investigators (Y.C. and Y.L.). A standardized collection form was applied for data extraction, collecting the following information: last name of the first author, country of the study, study design, publication year, collection time, sample size, age, outcome, and definition of osteoporosis and unadjusted effect estimates (hazard ratio, relative risk, or odds ratio) with CI. For studies provided both adjusted and unadjusted effect estimates (hazard ratio, relative risk, or odds ratio), the adjusted data were used for further analysis; OR is generally used in case–control studies and is better suited to low-frequency events than RR. OR was used as a common correlation measure in the included studies.

### 2.4. Quality assessment

Quality assessment was independently conducted by Y.C. and Y.L. and any discrepancies were resolved by B.D. The quality of the cohort and case–control studies was assessed using the Newcastle-Ottawa Scale (NOS), which is widely used in the quality assessment of case–control and cohort studies.^[16,17]^ NOS conducts a comprehensive evaluation of 3 aspects of the study: selection, comparability, and outcome (cohort studies) or exposure (case–control studies). A study can be awarded a maximum of 1 point for each numbered item within the selection and exposure categories. A maximum of 2 points can be assigned for comparability. The quality of the studies was assessed as follows: low quality, 0 to 3; moderate quality, 4 to 6; and high quality, 7 to 9.^[18]^ The quality of the cross-sectional studies was assessed using the 11-item checklist recommended by the Agency for Healthcare Research and Quality, which included the definition of information source, inclusion and exclusion criteria, time period and continuity to identify patients, blinding of personnel, assessments for quality assurance, confounding and missing data, and response rates and completeness of patients. An item would be scored “0” if it was answered “UNCLEAR” or “NO”; for the answer of “YES,” the item would get a score of “1.” The quality of the studies was scored as follows: low quality = 0 to 3, moderate quality = 4 to 7, and high quality = 8 to 11.^[19]^

### 2.5. Statistical analysis

State 15.1 software was used to analyze the data. Point estimates with standard errors were retrieved from each study and combined using the generic inverse variance method, as described by Der Simonian and Laird.^[20]^ Cochran *Q* test was used to determine statistical heterogeneity. This statistic was further supported by the I^2^ statistic, which quantifies the proportion of the total variation across studies from heterogeneity rather than coincidence. A value of I^2^ < 50% represents insignificant heterogeneity, I^2^ > 50% represents high heterogeneity, and a fixed-effects model or random-effects model was used separately to assess heterogeneity. Publication bias was assessed using Egger test when there were more than or equal to 10 included studies.

## 3. Results

### 3.1. Literature search

A total of 6368 articles were retrieved from the Embase, PubMed, Web of Science, and Cochrane Library databases, from which duplicated articles were discarded, leaving 4549 articles for title and abstract review. After reviewing the titles and abstracts, 2570 articles were excluded because they did not meet the eligibility criteria based on the study design and type of article, leaving 271 articles for full-length article review. A total of 245 articles were excluded because the outcome of interest was not reported, leaving 26 articles that fulfilled the eligibility criteria.

**Figure 1.**
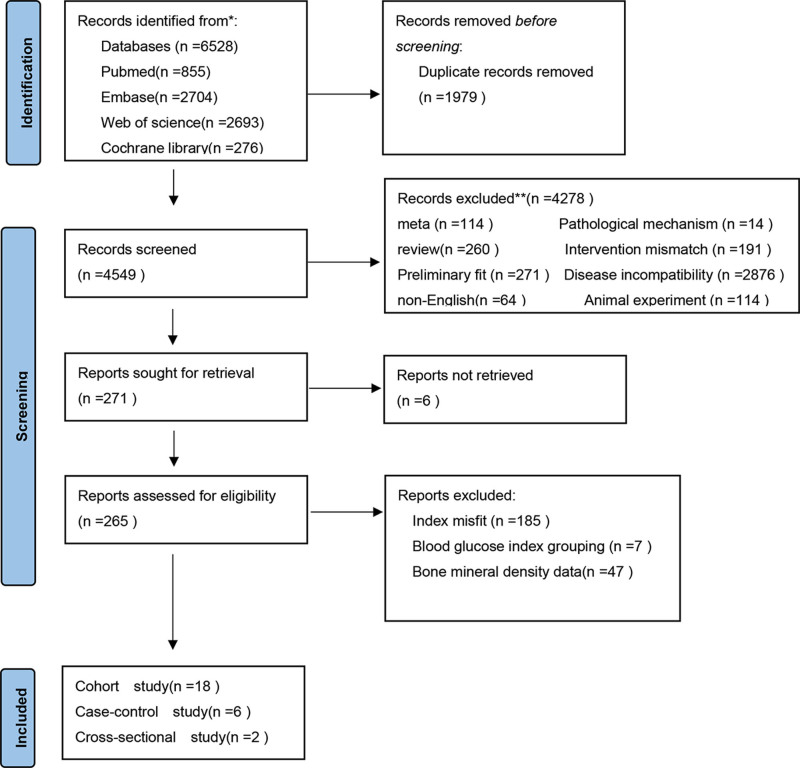
Flow chart of literature search, selection, and inclusion of studies for review.

Finally, 18 cohort studies,^[21–38]^ 6 case–control studies,^[8,39–43]^ and 2 cross-sectional studies,^[44,45]^ were included in the meta-analysis. Figure 1 shows the search methodology and selection process used in this study.

### 3.2. Study characteristics

Overall, the sample sizes of the included studies ranged from 283 to 6,546,366. The included studies were published between 2001 and 2022 and involved different ethnicities, including Asian and North American, European, and Oceanian populations, and included people aged 33 to 95 years. The assessment of the relationship between T2DM and osteoporosis or fracture has been inconsistent among studies, and detailed data on the main characteristics of these studies are shown in Table 1.

**Table 1:** Characteristics of the included studies.

Author	Year	Country	Research type	Collection time	Sample size	Age	Outcome	Total points
Wang et al	2022	China	Cohort	2018–2019	10,309	52.3 ± 11.57	Osteoporosis	7
van Hulten et al	2022	Holland	Prospective population-based cohort	November 2010–December 2017	2999	59.3 ± 8.51	Vertebral fracture	6
Schousboe et al	2022	Canada	Case–control		89,832	64.36 ± 11.15	Osteoporosis, hip fracture	7
Sarodnik et al	2022	United Kingdom	Retrospective cohort		248,656	62.9 ± 12.50	Fracture, hip fracture	8
Mesinovic et al	2021	Australia	Case–control	January 2005–June 2007	283	76.86 ± 5.46	Fracture, hip fracture	8
Lin et al	2021	Taiwan	Cohort	March 2002–October 2002	3331	55.08 ± 11.82	Osteoporosis	9
Davie et al	2021	United Kingdom	Prospective cohort		174,244	35–99	Fracture	8
Al Monaei et al	2021	Saudi Arabia	Cohort		1188	66.47 ± 8.77	Fracture	7
Park et al	2021	Korean	Population-based cohort	January 2009–December 2010	5,761,785	61.47 ± 8.62	Hip fracture	8
Lee et al	2021	Korean	Case–control	January 2014–December 2017	1130	63.22 ± 7.85	Osteoporosis	6
Ha et al	2021	Korean	Case–control	January 2009–December 2016	6,546,366	53.96 ± 10.27	Fracture, hip fracture, vertebral fracture	7
Tebé et al	2019	Spain	Cohort	January 2006–December 2013	126,035	72.14 ± 4.37	Fracture	7
Liu et al	2019	China	Cross-sectional		530	75.8 ± 10.98	Osteoporosis	10
Jiajue et al	2019	China	Cohort	March 2013–June 2014	982	61.76 ± 14,56	Fracture, vertebral fracture	8
Holm et al	2018	Danish	Historical cohort	2002–2012	6285	61.16 ± 11.64	Fracture, hip fracture	8
Kim et al	2017	Korean	Cross-sectional	January 2004–December 2010	51,330		Fracture, hip fracture, vertebral fracture	10
de Waard et al	2016	Netherlands	Cohort	November 2010–September 2013	1646	61.52 ± 6.24	Fracture	8
Rathmann et al	2015	German	Cohort	January 2000–December 2013	598,208	66.1 ± 12.2	Fracture, hip fracture	9
Schneider et al	2013	United States	Cohort		14,535	54.2 ± 5.73	Fracture	7
Lipscombe et al	2007	Canada	Retrospective cohort		598,812		Hip fracture	8
Janghorbani et al	2006	United States	Case–control		109,691	66.34 ± 9.44	Hip fracture	6
Bonds et al	2006	United States	Prospective cohort	October 1993–December 1998	93,405	63.58 ± 7.38	Fracture, hip fracture	7
Vestergaard et al	2005	Denmark	Case–control	January 2000–December 2000	498,617	43 ± 27	Fracture	9
Strotmeyer et al	2005	United States	Prospective cohort	1997–1998	2802		Fracture	6
Ottenbacher et al	2002	United States	Prospective cohort		1577	65–85	Hip fracture	7
Nicodemus et al	2001	United States	Prospective cohort		32,059		Hip fracture	4

### 3.3. Risk of bias assessment

Based on the NOS quality assessment and agency for Healthcare Research and Quality checklist, 21 studies were classified as high quality and 5 studies as moderate quality (Tables S2–S4, Supplemental Digital Content, http://links.lww.com/MD/O345). The comparability scores of the 2 medium-quality case–control studies were zero. In cohort and case–control studies, the controls were not community-based.

### 3.4. Meta-analyses

#### 3.4.1. Osteoporosis

**Figure 2.**
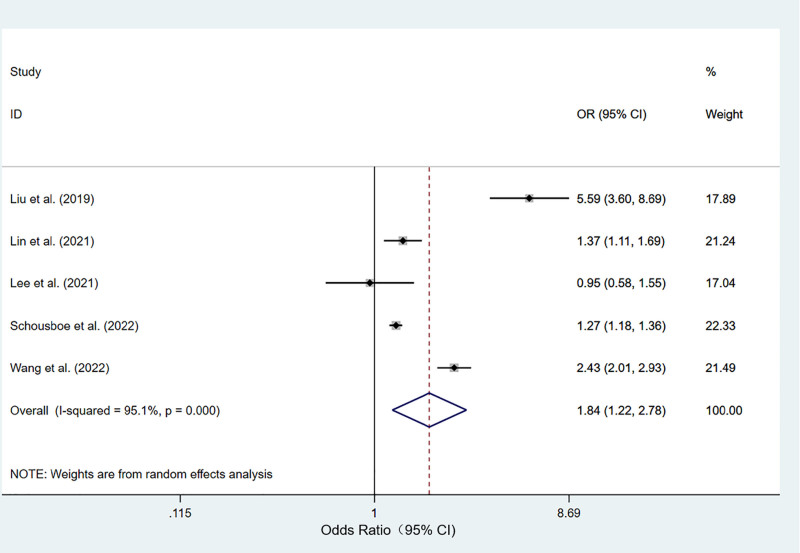
Meta-analysis of the relationship between type 2 diabetes mellitus and osteoporosis.

Five studies investigated the association between T2DM and osteoporosis.^[21,24,39,41,44]^ A random-effects model was used to analyze the effect size with modest heterogeneity among the studies (I^2^ = 95.10%, *P* < .001). The T2DM group had a significantly higher prevalence of osteoporosis than did the control group (OR: 1.841; 95% CI: 1.219–2.780; *P* = .004) (Fig. 2).

Subgroup analysis was performed for the variables of analysis method, study type, and geographical region because of the large heterogeneity among the studies. In the subgroup analysis, the T2DM group had a significantly higher prevalence of osteoporosis than the control group in the univariate analysis (OR: 2.365, 95% CI: 1.077–5.190; *P* = .032) and multivariate analysis (OR: 1.280; 95% CI: 1.197–1.369; *P* < .001). In the subgroup analysis for the study types, the T2DM group had a significantly higher prevalence of osteoporosis than the control group in cross-sectional studies (OR: 5.595, 95% CI: 3.601–8.693; *P* < .001), cohort studies (OR: 1.827, 95% CI: 1.043–3.200; *P* = .035), and case–control studies (OR: 1.221, 95% CI: 1.006–1.483; *P* < .001). In the subgroup analysis based on geographical region, the T2DM group had a significantly higher prevalence of osteoporosis than the control group in Asia (OR: 2.049; 95% CI: 1.168–3.598; *P* = .012) and North America (OR: 1.270; 95% CI: 1.183–1.363; *P* < .001) (Figures S1–S3, Supplemental Digital Content, http://links.lww.com/MD/O345).

#### 3.4.2. Fracture

**Figure 3.**
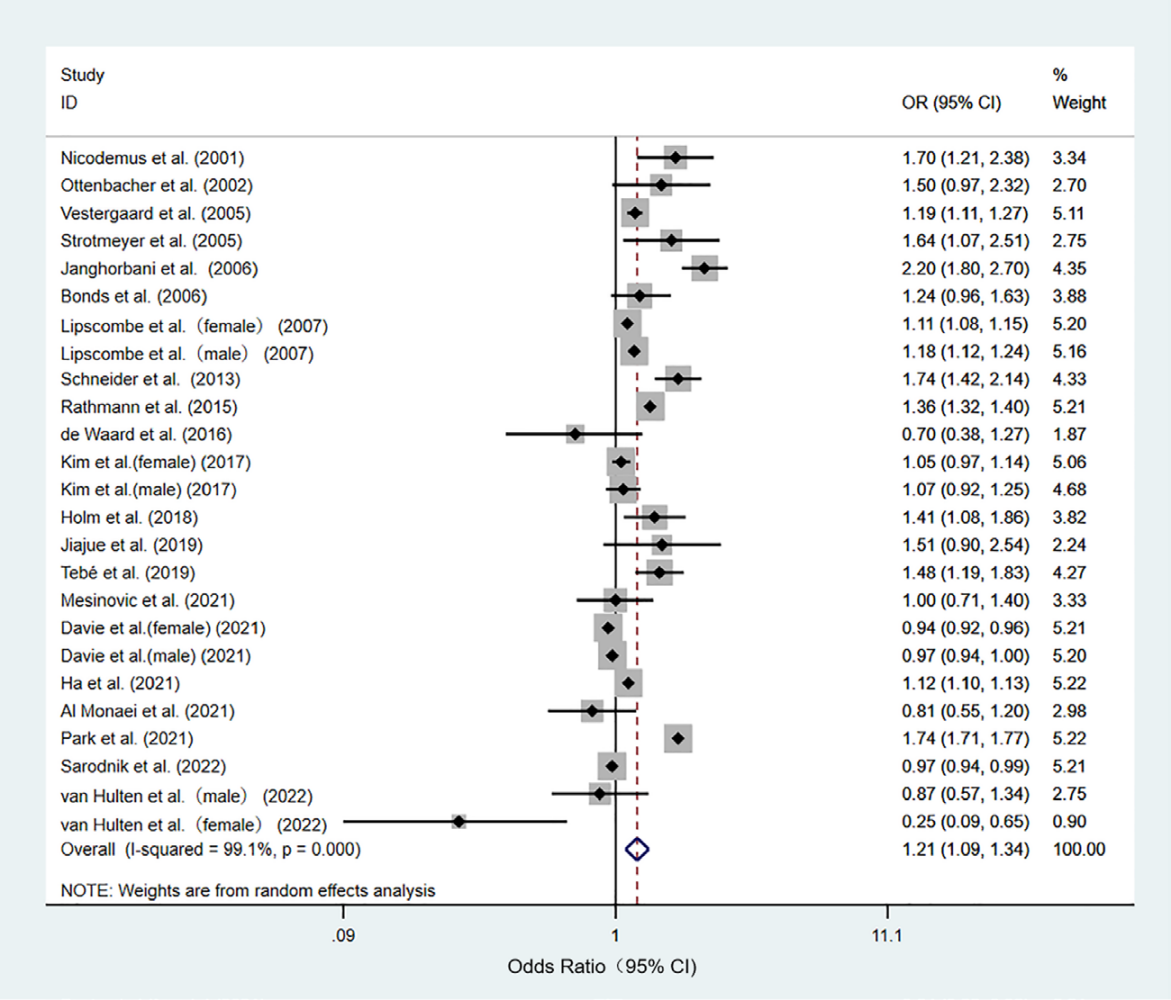
Meta-analysis of the relationship between type 2 diabetes mellitus and fractures.

Twenty-one studies investigated the association between T2DM and fractures.^[8,22–38,40,42,43,45]^ A random-effects model was used to analyze the effect size with modest heterogeneity among the studies (I^2^ = 99.1%, *P* < .001). The T2DM group had a significantly higher prevalence of fractures than the control group (OR: 1.21; 95% CI: 1.09–1.31; *P* < .001). In summary, there was a significant association between T2DM and fractures (Fig. 3).

Subgroup analysis was performed for the variables of the analysis method, study type, and geographical region because of the large heterogeneity observed. In the stratified analysis by analysis method, a statistically significant correlation was detected in multivariate analysis (OR: 1.23, 95% CI: 1.10–1.39; *P* = <.001); however, this association was not significant in univariate analysis (OR: 1.21, 95% CI: 0.89–1.40; *P* = .325). In the stratified analysis by study type, a statistically significant correlation was detected for cohort (OR: 1.20, 95% CI: 1.04–1.39; *P* = .012) and case–control studies (OR: 1.31, 95% CI: 1.10–1.55; *P* = .02); however, this association was not significant among cross-sectional studies (OR: 1.05, 95% CI: 0.98–1.13; *P* = .147). In the stratified analysis by geographical region, a statistically significant correlation was detected for North America (OR: 1.45, 95% CI: 1.26–1.65; *P* < .001), but this association was not significant among Oceania (OR: 1.000, 95% CI: 0.712–1.404; *P* = 1), Asia (OR: 1.18, 95% CI: 0.91–1.53; *P* = .207), or Europe (OR: 1.08, 95% CI: 0.95–1.23; *P* = .216) (Figures S4–S6, Supplemental Digital Content, http://links.lww.com/MD/O345).

According to the fracture sites described in the literature, 13 hip fractures and 4 vertebral fractures were found to be common fracture sites, and 15 had no other specific sites. Therefore, we conducted a subgroup analysis of hip and vertebral fractures. The results showed that hip fractures were significantly associated with T2DM (OR: 1.53, 95% CI: 1.33–1.75; *P* < .001), whereas vertebral fractures were not (OR: 0.98, 95% CI 0.89–1.07; *P* = .935 (Figure S7, Supplemental Digital Content, http://links.lww.com/MD/O345).

Based on the sex described in the literature, 11 articles were found to contain women and 7 contained males, and we performed a subgroup analysis for sex. T2DM was not associated with male sex (OR: 1.21, 95% CI: 0.98–1.49; *P* = .071); T2DM was associated with female sex (OR: 1.31, 95% CI: 1.05–1.64; *P* = .018) (Figure S8, Supplemental Digital Content, http://links.lww.com/MD/O345).

**Figure 4.**
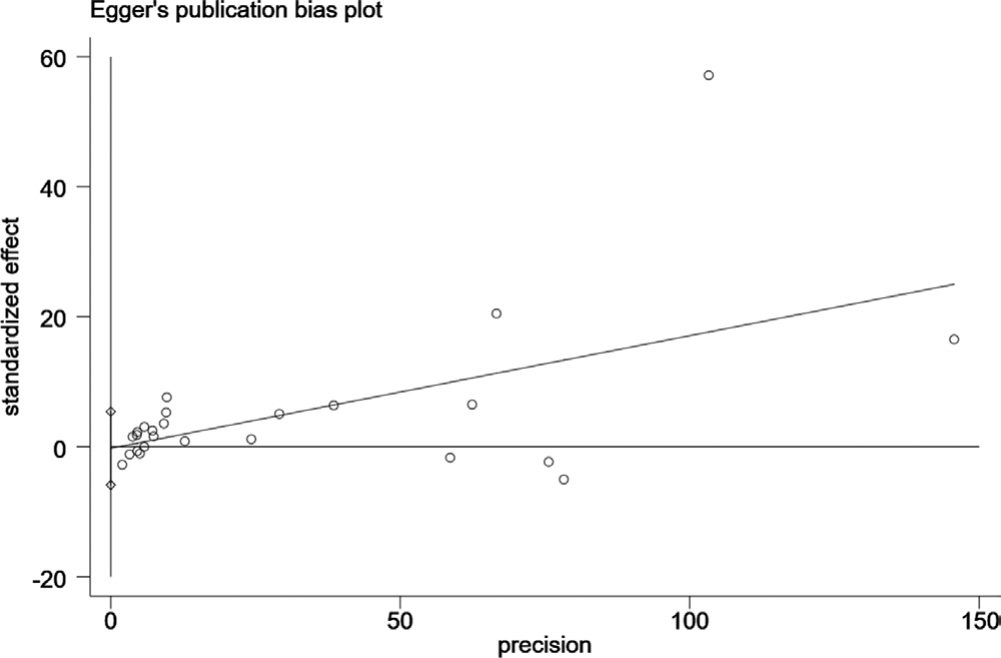
Publication bias in fractures.

Egger test was conducted because fractures were included in more than 10 studies. The *P*-value was .935, suggesting no significant publication bias (Fig. 4). Sensitivity analysis confirmed the robustness of our conclusions (Figures S9 and S10, Supplemental Digital Content, http://links.lww.com/MD/O345 and http://links.lww.com/MD/O345).

## 4. Discussion

We conducted a systematic review and meta-analysis to assess the impact of T2DM on fractures and osteoporosis, examining 26 large datasets with a total of 14,976,637 participants. The study showed that T2DM was significantly associated with the risk of osteoporosis, fracture, and hip fracture but not with the risk of vertebral fracture. Of the 5 articles cited regarding osteoporosis, 4 were consistent with the findings of this study. Additionally, 11 of the 21 articles cited on fractures aligned with the results presented in this paper, which could be observed across different study methods, analyses, and ethnicities, except for univariate analysis, cross-sectional analysis, and Europe, Asia, and Oceania analysis studies of fractures.

The underlying mechanism of the association between T2DM and osteoporosis/fracture risk is unclear, but there are some possible explanations. Complications of T2DM, including retinopathy and autonomic dysfunction, may contribute to bone fractures and osteoporosis by increasing the risk of falling.^[46]^ Diabetic microangiopathy^[47]^ and macroangiopathy^[48]^ which induce osteopenia, might also play a major role in the association between osteoporosis and diabetes, as some studies have found that retinopathy is slightly associated with osteopenia or osteoporosis.^[49]^ Alternatively, changes in the structure and quality of bone matrix components caused by the accumulation of inflammatory cytokines, advanced glycation end products, and oxidative stress have been suggested as explanations for changes in bone structure and bone strength properties.^[50,51]^

The univariate analysis showed that T2DM had no effect on fracture. Age, sex, geographical region, and other life differences all contributed to fractures. This suggests that when these factors are considered, T2DM has an impact on fracture risk. Cross-sectional studies do not deal with cause and effect, and the sample size was small; therefore, the results should be interpreted with caution. People in Oceania were selected from New South Wales, where medical security is better, the prevention and treatment of fractures is more complete, and the sample size is small; therefore, the results should be treated with caution. Among the Asian ethnic groups, the populations in the 3 articles with no significant correlation were selected from Beijing, South Korea, and Saudi Arabia, which had better medical facilities. One possible reason for the difference in fracture risk between the sexes in individuals with T2DM is the interaction between menopause and diabetes. Menopause has a greater effect on bone loss than chronological age, and bone loss is accelerated in postmenopausal women with diabetes.^[52,53]^

## 5. Limitations

Our analysis included an in-depth and extensive literature search of 26 studies, presented data of sufficient quality, and calculated outcome measures that were independent of the risk of research bias. However, our research has some limitations in the interpretation of the results. As a general defect in the meta-analysis of observational studies, we cannot rule out the possibility that certain residual factors might link T2DM with osteoporosis and fracture, such as environmental factors and medication use. Second, there have been few studies on vertebral fractures and osteoporosis. Finally, whether osteoporosis or fractures are caused by the disease itself, complications of the disease, or side effects of medications taken by some patients with diabetes has not been clarified. We recognize that this is a limitation; therefore, the results should be interpreted cautiously.

## 6. Conclusions

Through a meta-analysis of the association between diabetes and osteoporosis, we concluded that individuals with T2DM had a higher risk of osteoporosis and fractures than those without T2DM. These data suggest that patients with T2DM might be at risk of developing a comorbid diagnosis of osteoporosis and fractures, and should try to avoid risk factors and strengthen preventative measures against osteoporosis and fractures. More prospective cohort studies are needed to observe the effects of diabetes, its complications, and interventions on osteoporosis or fracture.

## Author contributions

**Conceptualization:** Yang Cao.

**Data curation:** Yue Li.

**Formal analysis:** Yang Cao.

**Funding acquisition:** Li Shen.

**Methodology:** Yang Cao, Yue Li.

**Resources:** Li Shen.

**Supervision:** Bo Dong, Li Shen.

**Writing – original draft:** Yang Cao.

**Writing – review & editing:** Bo Dong, Yue Li, Ying Liu.

## Supplementary Material


