# Supplementary material for: Association of type 2 diabetes with osteoporosis and fracture risk: A systematic review and meta-analysis
Source: Medicine (Baltimore). 2025 Feb 7;104(6):e41444. doi: 10.1097/MD.0000000000041444 (PMC11813021; doi:10.1097/MD.0000000000041444)

Table S1 Relevant databases retrieval record.

| Source: PubMed | | |
| --- | --- | --- |
| Search number | Search Details | Results |
| #13 | (((("Osteoporosis"[Mesh]) OR (((Osteoporosis[Title/Abstract]) OR (Osteoporoses[Title/Abstract])) OR (Bone Loss*[Title/Abstract]))) OR (("Fractures, Bone"[Mesh]) OR ((Bone Fracture*[Title/Abstract]) OR (Broken Bone*[Title/Abstract])))) AND (("Diabetes Mellitus, Type 2"[Mesh]) OR (((((Non-Insulin-Dependent Diabetes Mellitus[Title/Abstract]) OR (NIDDM[Title/Abstract])) OR (Noninsulin Dependent Diabetes Mellitus[Title/Abstract])) OR (Type 2 Diabetes[Title/Abstract])) OR (T2DM[Title/Abstract])))) AND (((((((observational) OR (cohort)) OR (case control)) OR (cross sectional)) OR (prospective)) OR (retrospective)) OR (follow up)) | 855 |
| #12 | ((("Osteoporosis"[Mesh]) OR (((Osteoporosis[Title/Abstract]) OR (Osteoporoses[Title/Abstract])) OR (Bone Loss*[Title/Abstract]))) OR (("Fractures, Bone"[Mesh]) OR ((Bone Fracture*[Title/Abstract]) OR (Broken Bone*[Title/Abstract])))) AND (("Diabetes Mellitus, Type 2"[Mesh]) OR (((((Non-Insulin-Dependent Diabetes Mellitus[Title/Abstract]) OR (NIDDM[Title/Abstract])) OR (Noninsulin Dependent Diabetes Mellitus[Title/Abstract])) OR (Type 2 Diabetes[Title/Abstract])) OR (T2DM[Title/Abstract]))) | 2,429 |
| #11 | (("Osteoporosis"[Mesh]) OR (((Osteoporosis[Title/Abstract]) OR (Osteoporoses[Title/Abstract])) OR (Bone Loss*[Title/Abstract]))) OR (("Fractures, Bone"[Mesh]) OR ((Bone Fracture*[Title/Abstract]) OR (Broken Bone*[Title/Abstract]))) | 311,593 |
| #10 | ("Fractures, Bone"[Mesh]) OR ((Bone Fracture*[Title/Abstract]) OR (Broken Bone*[Title/Abstract])) | 213,520 |
| #9 | ("Osteoporosis"[Mesh]) OR (((Osteoporosis[Title/Abstract]) OR (Osteoporoses[Title/Abstract])) OR (Bone Loss*[Title/Abstract])) | 123,808 |
| #8 | ("Diabetes Mellitus, Type 2"[Mesh]) OR (((((Non-Insulin-Dependent Diabetes Mellitus[Title/Abstract]) OR (NIDDM[Title/Abstract])) OR (Noninsulin Dependent Diabetes Mellitus[Title/Abstract])) OR (Type 2 Diabetes[Title/Abstract])) OR (T2DM[Title/Abstract])) | 228,316 |
| #7 | ((((((observational) OR (cohort)) OR (case control)) OR (cross sectional)) OR (prospective)) OR (retrospective)) OR (follow up) | 4,484,048 |
| #6 | (Bone Fracture*[Title/Abstract]) OR (Broken Bone*[Title/Abstract]) | 13,374 |
| #5 | "Fractures, Bone"[Mesh] | 206,759 |
| #4 | ((Osteoporosis[Title/Abstract]) OR (Osteoporoses[Title/Abstract])) OR (Bone Loss*[Title/Abstract]) | 108,454 |
| #3 | "Osteoporosis"[Mesh] | 62,407 |
| #2 | ((((Non-Insulin-Dependent Diabetes Mellitus[Title/Abstract]) OR (NIDDM[Title/Abstract])) OR (Noninsulin Dependent Diabetes Mellitus[Title/Abstract])) OR (Type 2 Diabetes[Title/Abstract])) OR (T2DM[Title/Abstract]) | 176,083 |
| #1 | "Diabetes Mellitus, Type 2"[Mesh] | 168,402 |

| Source: Embase | | |
| --- | --- | --- |
| No. | Query | Results |
| #13 | #7 AND #12 | 2704 |
| #12 | #8 AND #11 | 7811 |
| #11 | #9 OR #10 | 578996 |
| #10 | #5 OR #6 | 443976 |
| #9 | #3 OR #4 | 204280 |
| #8 | #1 OR #2 | 374342 |
| #7 | observational OR cohort OR (case AND control) OR (cross AND sectional) OR prospective OR retrospective OR (follow AND up) | 6466986 |
| #6 | 'broken bone*':ab,ti OR fracture:ab,ti | 251294 |
| #5 | 'fracture'/exp | 383978 |
| #4 | osteoporoses:ab,ti OR osteoporosis:ab,ti OR 'bone loss*':ab,ti | 152196 |

| | | |
|---|---|---|
| #3 | 'osteoporosis'/exp | 152280 |
| #2 | 'non-insulin-dependent diabetes mellitus':ab,ti OR niddm:ab,ti OR 'noninsulin dependent diabetes mellitus':ab,ti OR 'type 2 diabetes':ab,ti OR t2dm:ab,ti | 260505 |
| #1 | 'non insulin dependent diabetes mellitus'/exp | 327229 |

Source: Cochrane Library

| ID | Search | Results |
|---|---|---|
| #1 | MeSH descriptor: [Diabetes Mellitus, Type 2] explode all trees | 22903 |
| #2 | (Non-Insulin-Dependent Diabetes Mellitus):ti,ab,kw OR (NIDDM):ti,ab,kw OR (Noninsulin Dependent Diabetes Mellitus):ti,ab,kw OR (Type 2 Diabetes):ti,ab,kw OR (T2DM):ti,ab,kw | 58683 |
| #3 | MeSH descriptor: [Osteoporosis] explode all trees | 5754 |
| #4 | (Osteoporosis):ti,ab,kw OR (Osteoporoses):ti,ab,kw OR (Bone Loss*):ti,ab,kw | 20254 |
| #5 | MeSH descriptor: [Fractures, Bone] explode all trees | 8166 |
| #6 | (Bone Fracture*):ti,ab,kw OR (Broken Bone*):ti,ab,kw | 12694 |
| #7 | #1OR#2 | 58684 |
| #8 | #3OR#4 | 20254 |
| #9 | #5OR#6 | 15350 |
| #10 | #8OR#9 | 29640 |
| #11 | #7AND#10 | 585 |
| #12 | (observational) OR (cohort) OR (case control) OR (cross sectional) OR (prospective) | 375043 |
| #13 | (retrospective) OR (follow up) | 326698 |
| #14 | #12OR#13 | 592660 |
| #15 | #14AND#11 | 276 |

Source: Web of science

| No. | Query | Results |
|---|---|---|
| #88 | #83 AND #75 and Preprint Citation Index (Exclude – Database) | 2693 |
| #83 | #77 AND #62 and Preprint Citation Index (Exclude – Database) | 10991 |
| #77 | #68 OR #53 and Preprint Citation Index (Exclude – Database) | 1575452 |
| #75 | ((((((TS=(observational)) OR TS=(cohort)) OR TS=(case control)) OR TS=(cross sectional)) OR TS=(prospective)) OR TS=(retrospective)) OR TS=(follow up) and Preprint Citation Index (Exclude – Database) | 9083134 |
| #68 | (TS=(Fractures)) OR TS=(Broken Bone*) and Preprint Citation Index (Exclude – Database) | 1301673 |
| #62 | ((((TS=(Non-Insulin-Dependent Diabetes Mellitus)) OR TS=(NIDDM)) OR TS=(Noninsulin Dependent Diabetes Mellitus)) OR TS=(Type 2 Diabetes)) OR TS=(T2DM) and Preprint Citation Index (Exclude – Database) | 451695 |
| #53 | ((TS=(Osteoporosis)) OR TS=(Osteoporoses)) OR TS=(Bone Loss*) and Preprint Citation Index (Exclude – Database) | 365698 |

Table S2 Newcastle-Ottawa Scale (cohort) for eighteen studies included in this meta-analysis.

| Study | Selection | | | | Comparability | Outcome | | | Quality scores |
|---|---|---|---|---|---|---|---|---|---|
| | Representativeness of the exposed cohort | Selection of the nonexposed cohort | Ascertainment of exposure | Demonstration that outcome of interest was not present at start of study | Comparability of cohorts on the basis of the design or analysis | Assessment of outcome | Was follow-up long enough for outcomes to occur | Adequacy of follow up of cohorts | |
| Wang 2022 | **1** | **1** | **1** | **0** | **1** | **1** | **1** | **1** | 7 |
| van Hulten2022 | **0** | **1** | **1** | **0** | **1** | **1** | **1** | **1** | 6 |
| Sarodnik 2022 | **1** | **1** | **1** | **0** | **2** | **1** | **1** | **1** | 8 |
| Lin2021 | **1** | **1** | **1** | **1** | **2** | **1** | **1** | **1** | 9 |
| Davie2021 | **1** | **1** | **1** | **0** | **2** | **1** | **1** | **1** | 8 |
| AlMonaei 2021 | **1** | **1** | **1** | **0** | **1** | **1** | **1** | **1** | 7 |
| Park 2021 | **1** | **1** | **1** | **0** | **2** | **1** | **1** | **1** | 8 |
| Jiajue2019 | **1** | **1** | **1** | **0** | **2** | **1** | **1** | **1** | 8 |
| Tebé2019 | **1** | **1** | **1** | **0** | **1** | **1** | **1** | **1** | 7 |
| Holm2018 | **1** | **1** | **1** | **0** | **2** | **1** | **1** | **1** | 8 |
| de Waard2016 | **1** | **1** | **1** | **0** | **2** | **1** | **1** | **1** | 8 |
| Rathmann2015 | **1** | **1** | **1** | **1** | **2** | **2** | **1** | **1** | 9 |
| Schneider 2013 | **1** | **1** | **1** | **0** | **2** | **1** | **1** | **0** | 7 |
| Lipscombe 2007 | **1** | **1** | **1** | **0** | **2** | **1** | **1** | **1** | 8 |
| Bonds2006 | **1** | **1** | **1** | **0** | **2** | **1** | **1** | **0** | 7 |
| Strotmeyer 2005 | **0** | **1** | **1** | **0** | **2** | **1** | **1** | **0** | 6 |
| Ottenbacher2002 | **1** | **1** | **1** | **0** | **2** | **1** | **1** | **0** | 7 |
| Nicodemus 2001 | **0** | **1** | **0** | **0** | **2** | **0** | **1** | **0** | 4 |

Table S3 Newcastle-Ottawa Scale (case-control) for six studies included in this meta-analysis.

| Study | Selection | | | | Comparability | Exposure | | | Overall score |
|---|---|---|---|---|---|---|---|---|---|
| | Is the case definition adequate? | Representativeness of the cases | Selection of controls | Definition of Controls | Comparability of cohorts on the basis of the design or analysis | Ascertainment of exposure | Same method of ascertainment for cases and controls | non-response rate | |
| Schousboe 2022 | **1** | **1** | **0** | **1** | **2** | **1** | **1** | **0** | 7 |
| Mesinovic 2021 | **1** | **1** | **1** | **1** | **2** | **1** | **1** | **0** | 8 |
| Lee2021 | **0** | **1** | **0** | **0** | **2** | **1** | **1** | **1** | 6 |
| Ha2021 | **1** | **1** | **0** | **0** | **2** | **1** | **1** | **1** | 7 |
| Janghorbani2006 | **0** | **1** | **1** | **1** | **2** | **0** | **1** | **0** | 6 |
| Vestergaard2005 | **1** | **1** | **1** | **1** | **2** | **1** | **1** | **0** | 9 |

Table S4 Agency for healthcare research and quality (AHRQ) checklist (cross-sectional) for 2 studies included in this meta-analysis.

| Study | Define the source of information (survey, record review) 1 | List inclusion and exclusion criteria for exposed and unexposed subjects (cases and controls) or refer to previous publications. 2 | Indicate the time period used for identifying patients. 3 | Indicate whether or not the subjects were consecutive if not population based. 4 | Indicate if the evaluators of subjective components of study were masked to other aspects of the status of the participants. 5 | Describe any assessments undertaken for quality assurance purposes (e.g., test/retest of primary outcome measurements). 6 | Explain any patient exclusions from the analysis. 7 | Describe how confounding was assessed and/or controlled 8 | If applicable, explain how missing data were handled in the analysis. 9 | Summarize the patient response rates and completeness of data collection. 10 | Clarify what follow-up, if any, was expected and the percentage of patients for which incomplete data or follow-up was obtained. 11 | scores |
|---|---|---|---|---|---|---|---|---|---|---|---|---|
| Liu2019 | **1** | **1** | **1** | **1** | **1** | **1** | **1** | **0** | **1** | **1** | **1** | 10 |
| Kim2017 | **1** | **1** | **1** | **1** | **1** | **1** | **0** | **1** | **1** | **1** | **1** | 10 |

Fig. S1 Subgroup analysis of osteoporosis by analysis

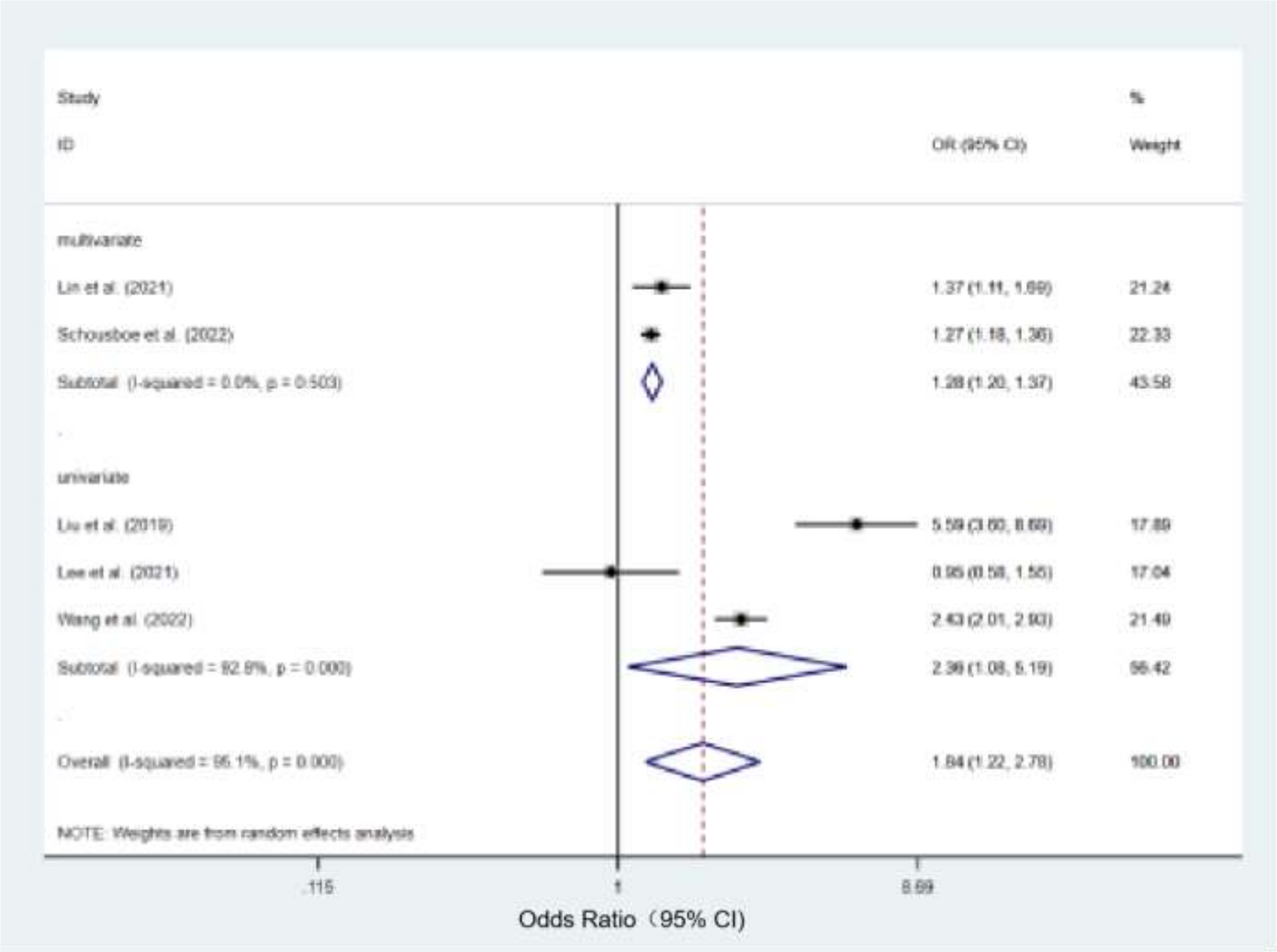

Fig. S2 Subgroup analysis of osteoporosis according to research type

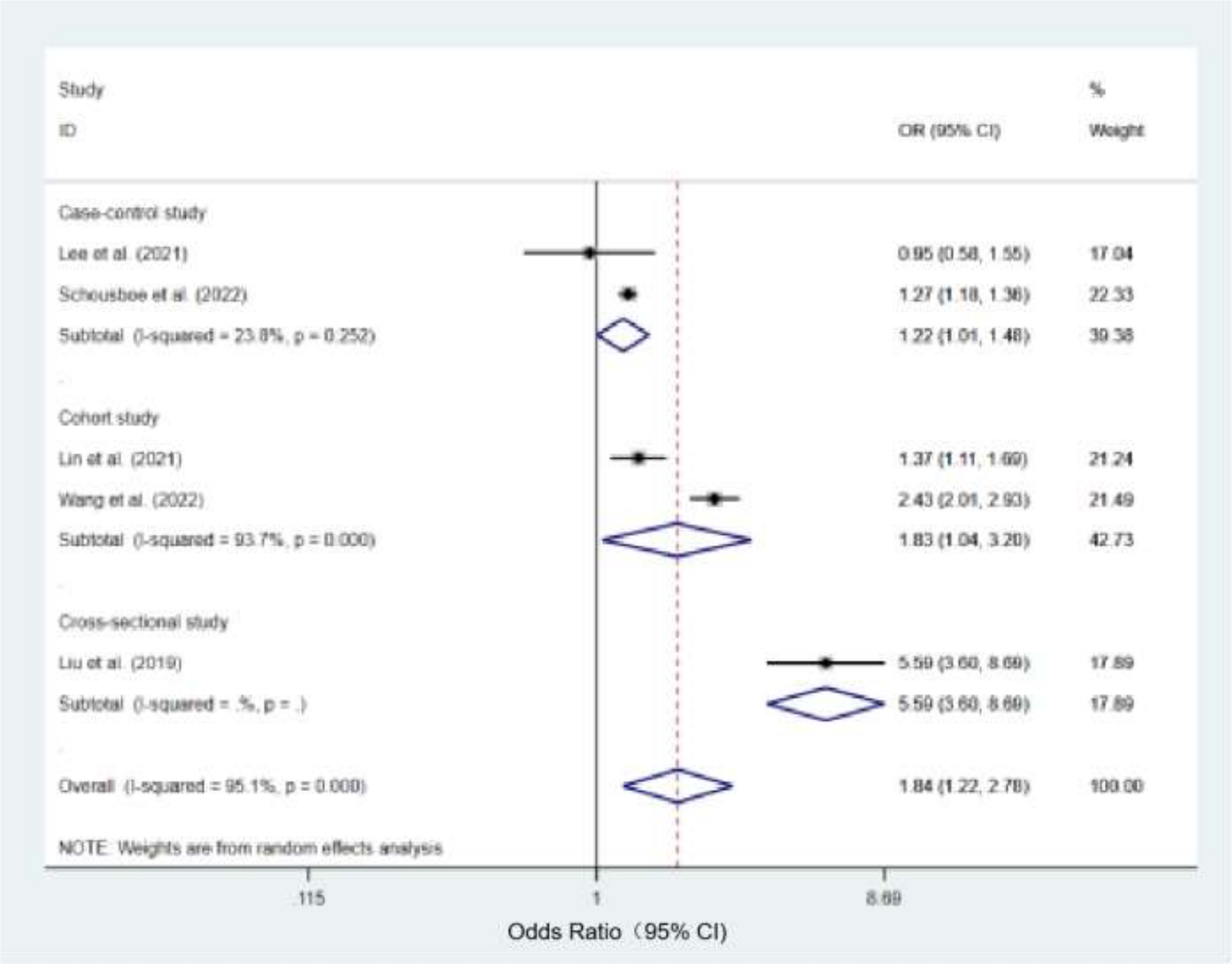

Fig. S3 Subgroup analysis of osteoporosis according to geographical region

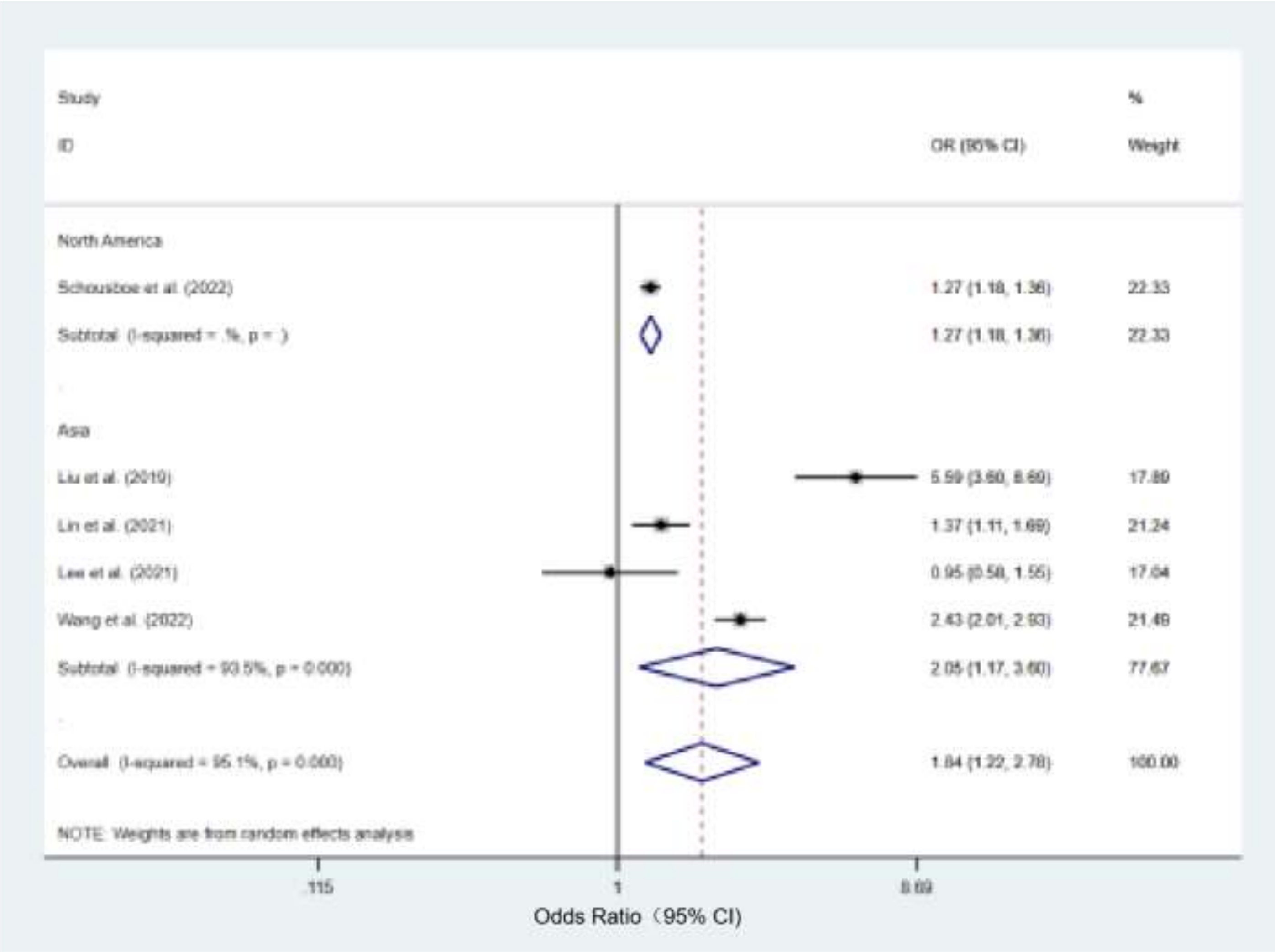

Fig. S4 Subgroup analysis of fractures by means of analysis

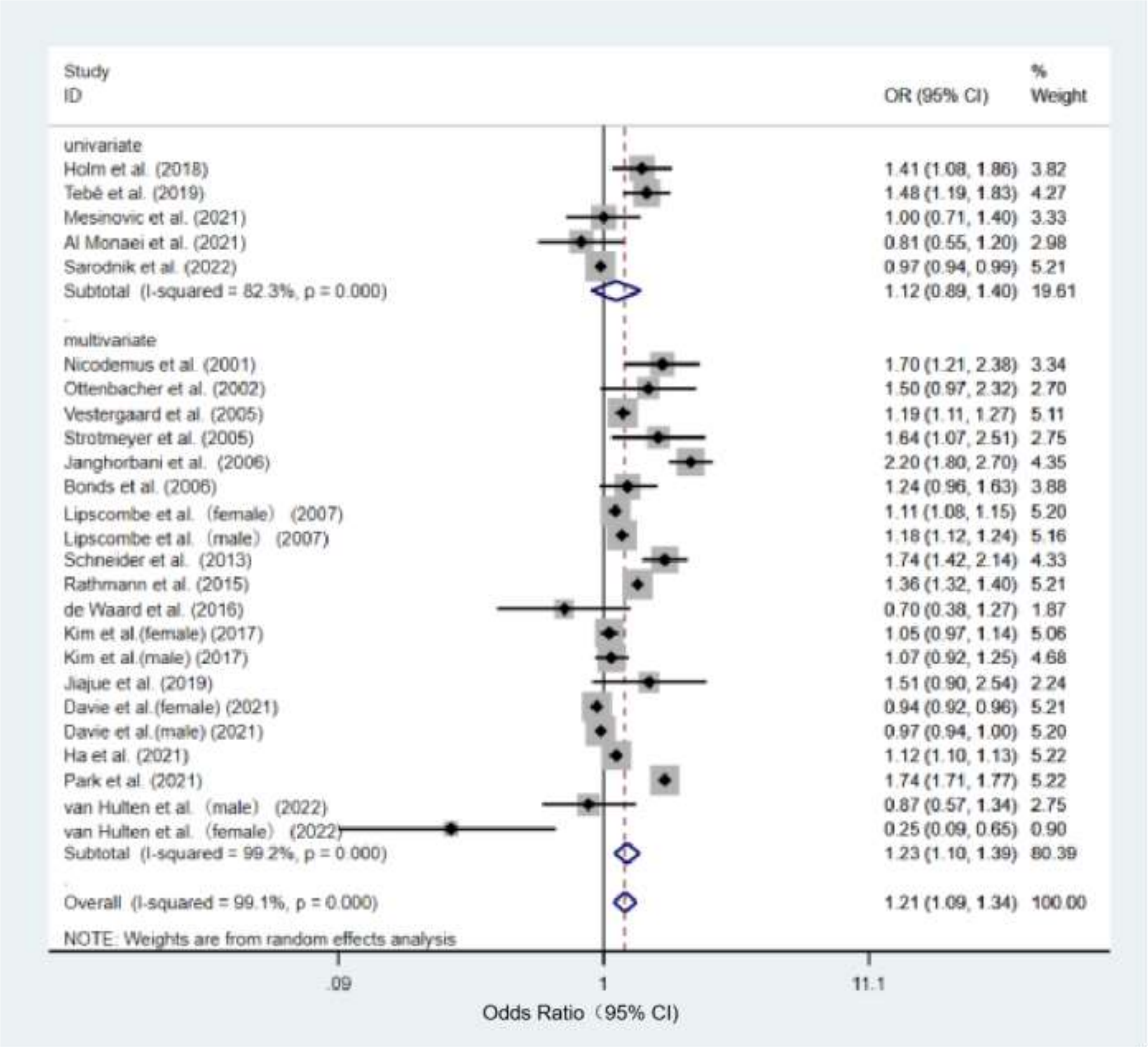

Fig. S5 Subgroup analysis of fractures according to research type.

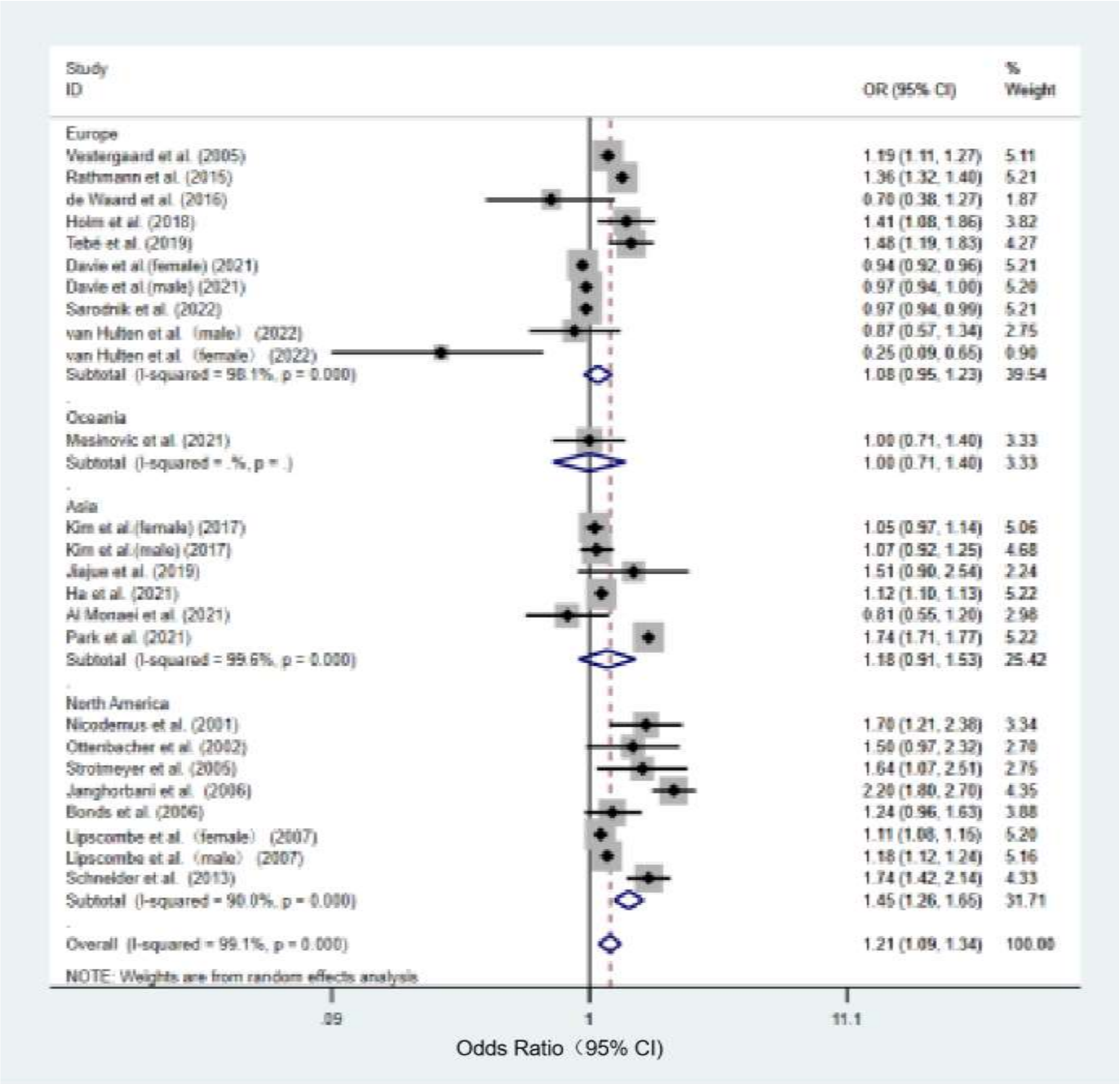

Fig. S6 Subgroup analysis of fractures by geographical region

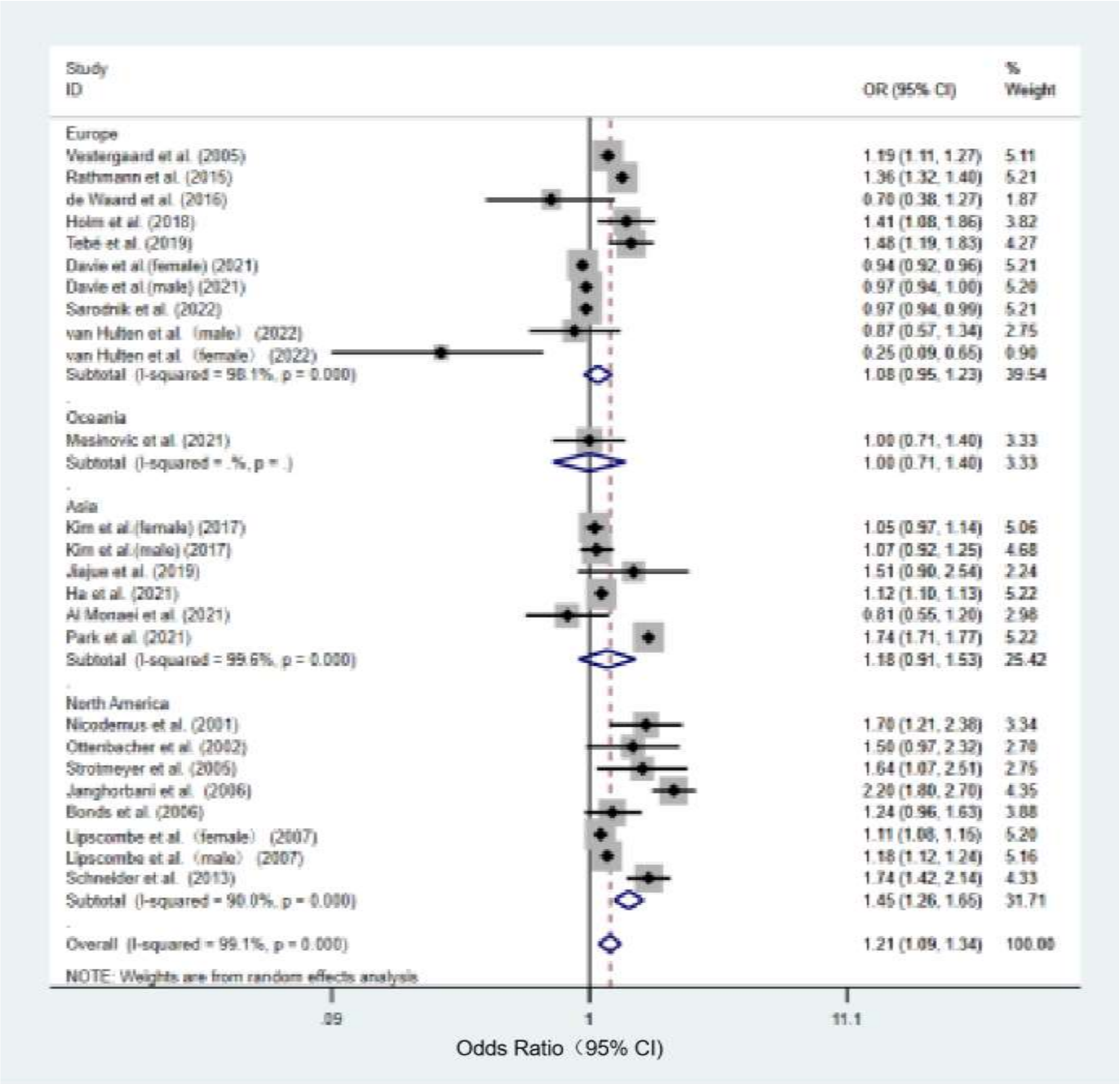

Fig. S7 Subgroup analysis of fracture-by-fracture sites (hip and vertebral fractures).

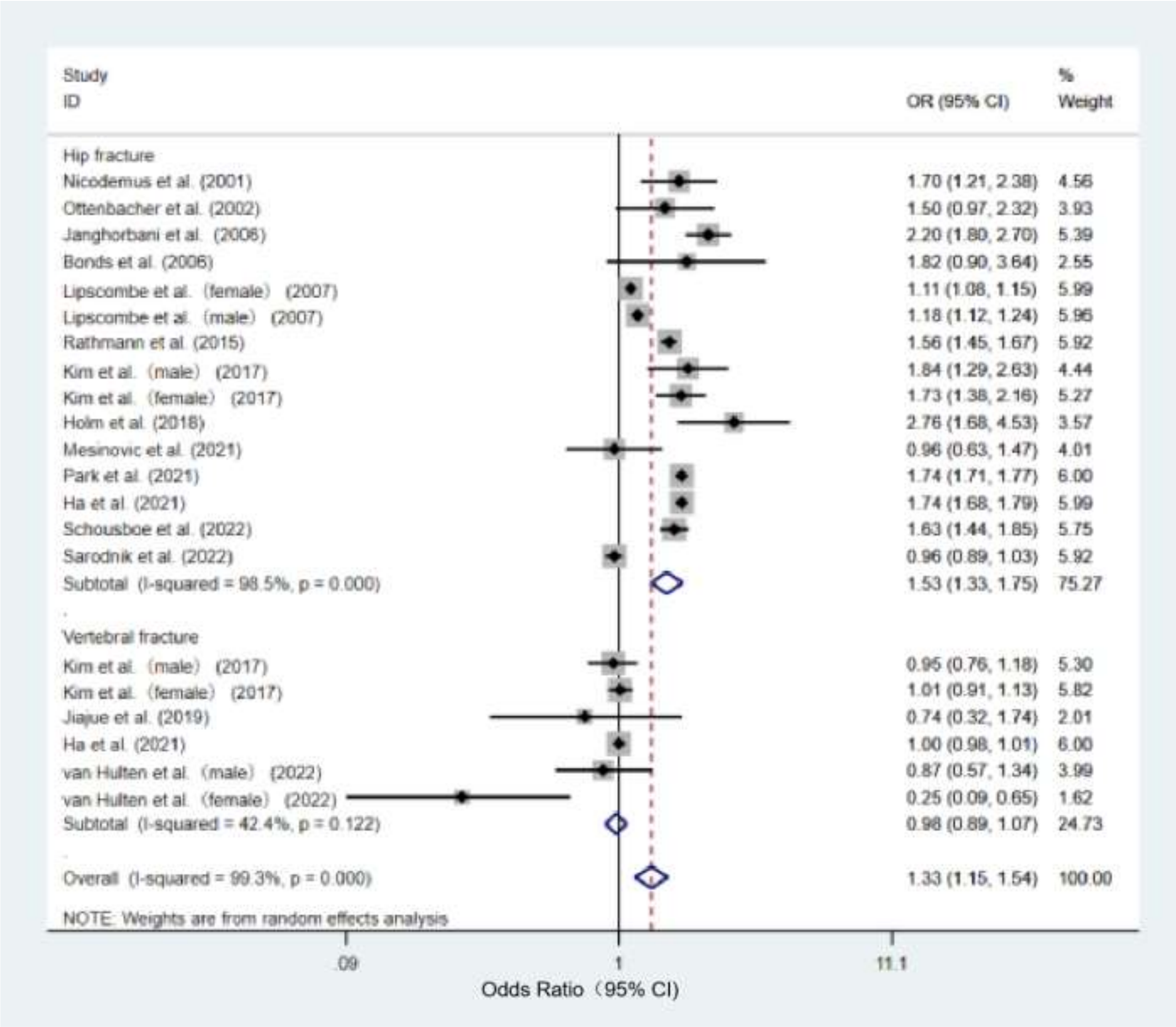

Fig. S8 Subgroup analysis of fractures by sex

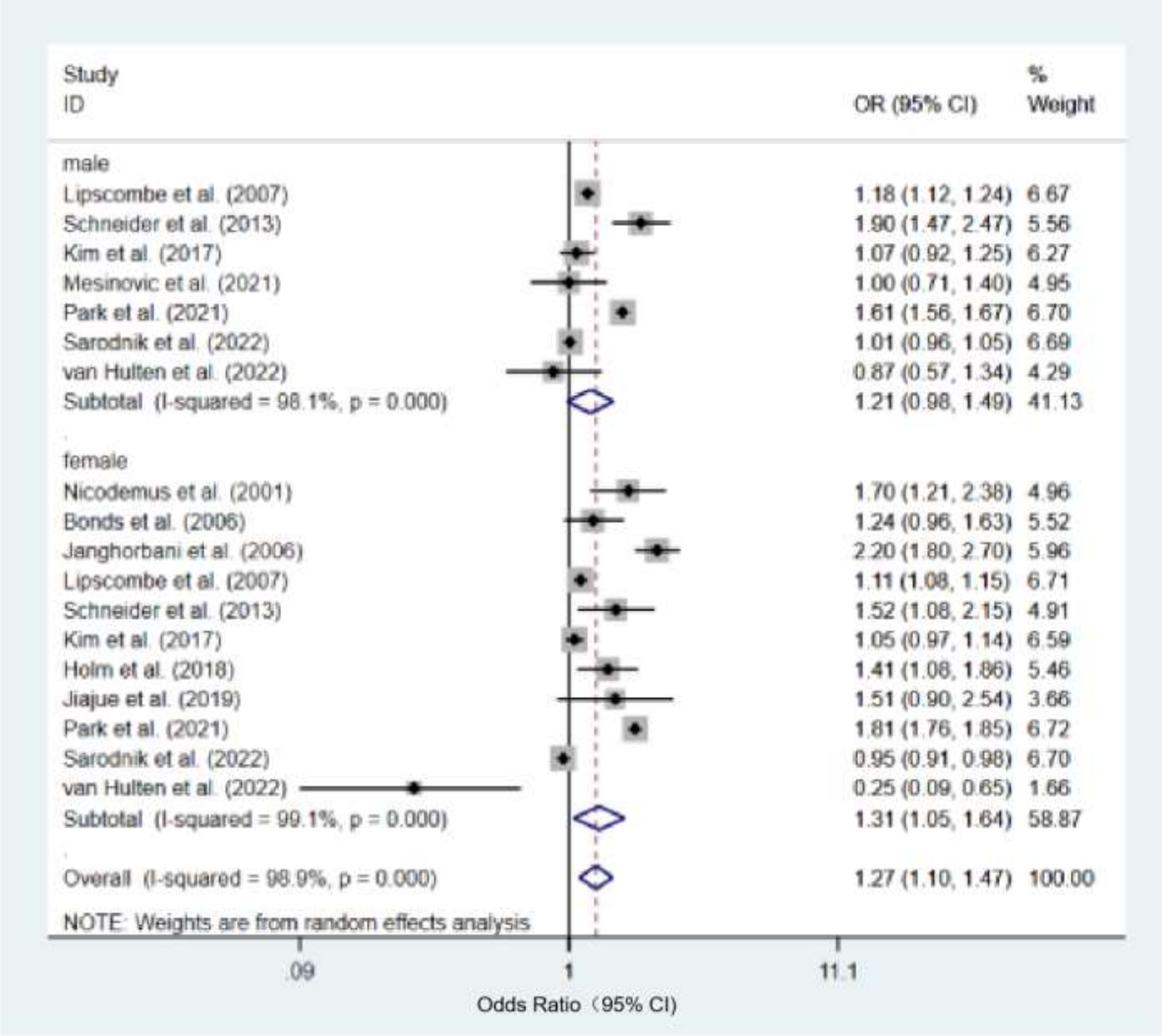

Fig. S9: Sensitivity analysis of osteoporosis.

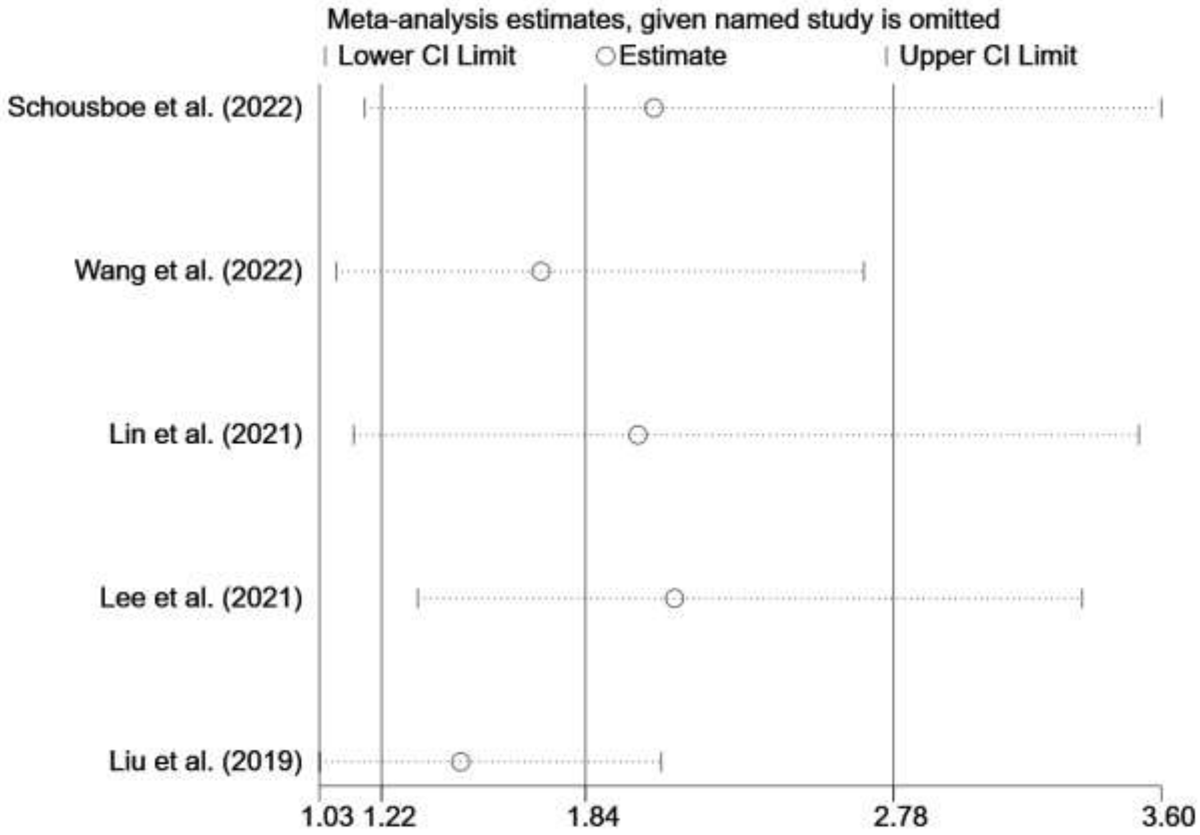

Fig. S10 Sensitivity analysis of fractures

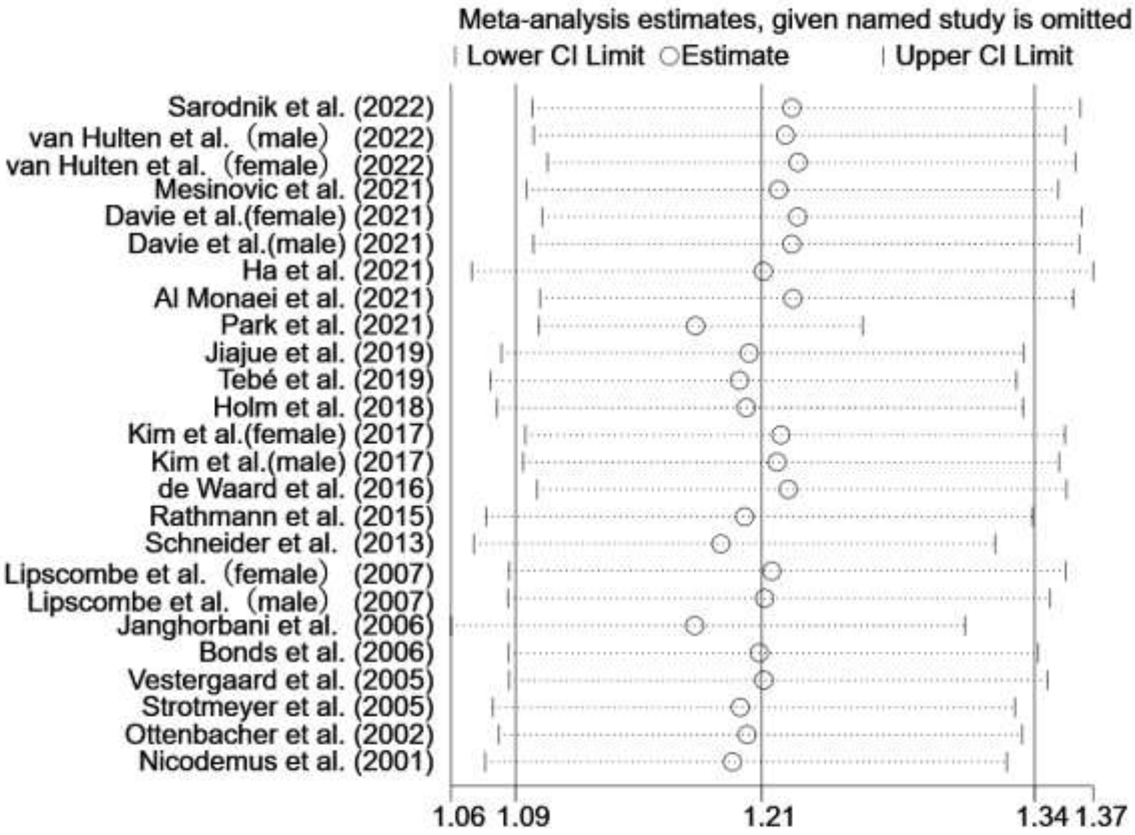

Supplement: Supplementary file 1 [file medi-104-e41444-s001.pdf]
